# Sustainability in Membrane Technology: Membrane Recycling and Fabrication Using Recycled Waste

**DOI:** 10.3390/membranes14020052

**Published:** 2024-02-12

**Authors:** Noman Khalid Khanzada, Raed A. Al-Juboori, Muzamil Khatri, Farah Ejaz Ahmed, Yazan Ibrahim, Nidal Hilal

**Affiliations:** NYUAD Water Research Center, New York University Abu Dhabi, Abu Dhabi P.O. Box 129188, United Arab Emirates; noman.khanzada@nyu.edu (N.K.K.); raa9914@nyu.edu (R.A.A.-J.); muzamilkhatri@nyu.edu (M.K.); farah.ahmed@nyu.edu (F.E.A.); ymi212@nyu.edu (Y.I.)

**Keywords:** membrane recycling, waste-derived membranes, sustainable membranes, waste recycling

## Abstract

Membrane technology has shown a promising role in combating water scarcity, a globally faced challenge. However, the disposal of end-of-life membrane modules is problematic as the current practices include incineration and landfills as their final fate. In addition, the increase in population and lifestyle advancement have significantly enhanced waste generation, thus overwhelming landfills and exacerbating environmental repercussions and resource scarcity. These practices are neither economically nor environmentally sustainable. Recycling membranes and utilizing recycled material for their manufacturing is seen as a potential approach to address the aforementioned challenges. Depending on physiochemical conditions, the end-of-life membrane could be reutilized for similar, upgraded, and downgraded operations, thus extending the membrane lifespan while mitigating the environmental impact that occurred due to their disposal and new membrane preparation for similar purposes. Likewise, using recycled waste such as polystyrene, polyethylene terephthalate, polyvinyl chloride, tire rubber, keratin, and cellulose and their derivates for fabricating the membranes can significantly enhance environmental sustainability. This study advocates for and supports the integration of sustainability concepts into membrane technology by presenting the research carried out in this area and rigorously assessing the achieved progress. The membranes’ recycling and their fabrication utilizing recycled waste materials are of special interest in this work. Furthermore, this study offers guidance for future research endeavors aimed at promoting environmental sustainability.

## 1. Introduction

The escalating issue of water shortage has emerged as a worldwide threat owing to its crucial role in maintaining environmental sustainability and ecological systems [1,2,3,4,5,6,7]. Desalination and wastewater reclamation utilizing membrane-based technologies are regarded as effective strategies for mitigating water scarcity [8]. In contrast to traditional treatment methods, membrane technology presents a multitude of benefits, such as superior quality of treated water, compact and resilient construction, economical operation, and maintenance expenses, and more [9,10,11]. Microfiltration (MF), ultrafiltration (UF), nanofiltration (NF), reverse osmosis (RO), forward osmosis (FO), and membrane distillation (MD) are some of the classifications of membranes that are determined by the separation performance that is linked to the morphological structure and operational principle of the process [12]. The common polymeric membrane materials used in MF, UF, and MD processes include polyether sulfone (PES), polyvinylidene fluoride (PVDF), polysulfone (PSF), polytetrafluoroethylene (PTFE), etc. [13,14,15], whereas for NF and RO, thin film composite (TFC) and thin film nanocomposite (TFN) polyamide (PA) membranes are dominating the market [16,17,18]. Forecasts indicate that by 2024, the worldwide membrane market will expand from USD 5.4 billion in 2019 to USD 8.3 billion [19].

These polymeric membranes derived from fossil fuel inevitably encounter their end of life due to various factors, including the deposition of unanticipated fouling, mechanical/chemical persuaded defects, etc. As a consequence, appropriate disposal and replacement procedures become imperative [20,21]. The annual disposal of end-of-life spiral-wound membrane elements from desalination facilities around the world was estimated by Landaburu-Aguirre et al. to exceed 840,000 (or 14,000 tons in weight) [20]. In general, the discarded membranes are disposed of in accordance with the waste management regulations of each country, which stipulate their incineration of landfill disposal [21,22,23]. Nevertheless, the environmental impact of both incineration and landfills remains detrimental. For instance, the landfill degradation process for a frequently employed 8-inch spiral-wound RO membrane element (length 1 m; weight 18 kg) spanned several years. In regard to incineration, insufficient waste management and control may result in the production of greenhouse gases and other detrimental byproducts [20]. The plastic components of the end-of-life RO membrane modules are prone to producing toxic and carcinogenic byproducts when incinerated, posing a risk to human health. This emphasizes the criticality of environmentally sustainable disposal approaches that adhere to the circular economy and sustainability principles on a global scale [24].

In parallel to it, numerous industrial processes and everyday activities inevitably result in waste production, with the quantity of waste produced rising in direct correlation with product demand from increasing population [25,26,27,28,29]. Polystyrene foam (used in packaging and disposable item manufacturing), polyethylene terephthalate (used in making bottles, automobile parts, electronic items, cosmetics, other packaging, etc.), polyvinyl chloride (used in making automotive spare parts, pipelines, cables, home items, etc.), tire rubber, keratin (generated from the animal body parts such as feathers, hairs, nails, hooves, etc.), and cellulose and its derivates (produced from oil palm empty fruit bunches, kapok fiber, cotton microdust waste released during the spinning process, waste paper, waste cigarettes, etc.) are examples of proportionally generated waste [30,31,32]. Not only does the inadequate disposal and management of this waste give rise to significant environmental issues but it also has adverse effects on human health [33,34]. Given the magnitude of these challenges, it is imperative that scientists investigate potential remedies, which may involve the development of cleaner technologies and the implementation of more sustainable resources. Greater emphasis has been placed on waste management, product life cycle assessment, and circular economy strategies as a result of the alarming level of waste production [35,36]. In order to address economic and environmental issues and adhere to the recently implemented regulations on sustainable economies, circular methods have been suggested in industrial sectors to facilitate the reuse of waste materials that may be transformed into raw resources [37,38]. As a consequence, contemporary manufacturing industries are reorienting their focus toward material sourcing and minimizing the consumption of basic materials [39,40,41].

Recycling membranes and utilizing recovered waste material for their fabrication can effectively address both resource and water scarcity challenges while concurrently reducing the generated waste amount and its associated environmental impact occurring upon their disposal [30,42,43,44]. The suggested approach not only makes the membrane-based water treatment process sustainable but it also further aligns it with the concept of a circular economy. This study aims to promote sustainability awareness and its inclusion in membrane technology. The review provides a summary of the recently conducted work related to membrane recycling and their fabrication using recycled waste materials, in addition to the critical evaluation of the scientific literature. It further intends to provide guidance and research direction for the broad membrane community as well as industries practicing desalination and water/wastewater treatment.

## 2. Polymer and Polymeric Membrane Recycling

Membrane recycling refers to the reuse of polymeric membranes (that are nearing the end of their lifespan) after undergoing chemical treatment for similar or upgraded/downgraded operation, whereas polymer recycling refers to the complete membrane deformation to recover polymer for re-preparation of membrane or other applications. In the case of membranes that have not suffered any significant damage, a number of different techniques, such as upcycling, downcycling, and regeneration, may be utilized, depending on the particular conditions. In the event of significant membrane damage, it is advisable to dissolve the membrane in organic solvents and then prepare a new membrane. Both MF and UF membranes can undergo either upcycling or regeneration at the end of their lifespan, depending on the user’s preferred requirement. Likewise, the NF or RO membranes that have reached the end of their useful life can be either downcycled or regenerated to meet the specific needs of the user. The sections below describe the adopted methods and strategies utilized for making membrane processes sustainable and cost-effective.

### 2.1. Polymeric Membrane Regeneration

**Figure 1:**
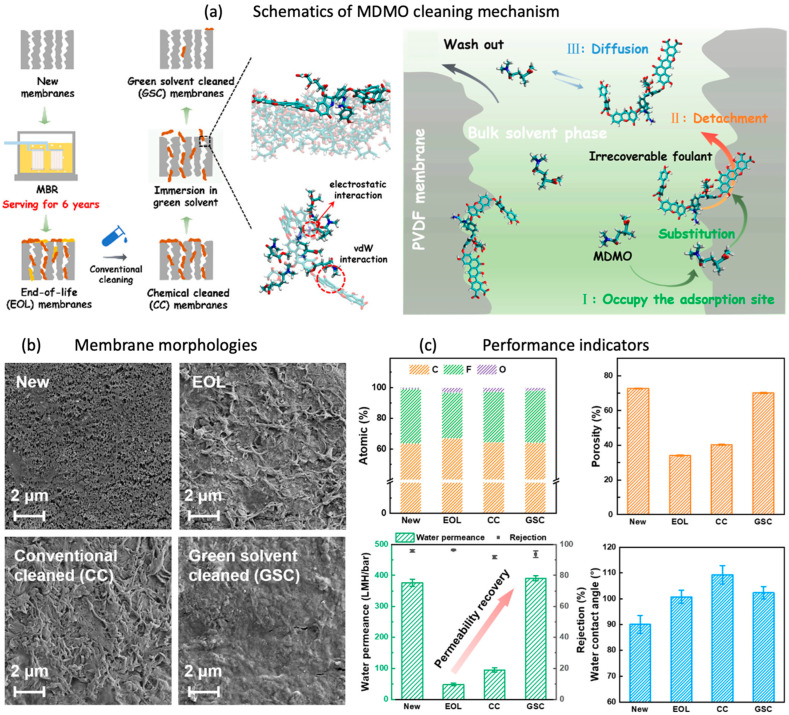
End-of-life membrane regeneration using green solvent: (**a**) schematics of MDMO cleaning mechanism; (**b**,**c**) SEM images and performance of the pristine, end-of-life, conventionally cleaned, and green-solvent-cleaned membrane [47]. Reprinted with copyright permission.

Membrane regeneration is the process of recycling membranes to regain pristine properties. The fouling deposition over the membrane during operation provides a hindrance to water permeance, thus resulting in enhanced trans-membrane pressure, high energy utilization, and permeate quality deterioration [45]. For successful membrane regeneration, the choice of solvent plays a vital role. It is important to choose a solvent that can effectively remove the foulants from the membrane’s adsorption sites by interacting strongly with the membrane matrix [46]. Methyl-5-(dimethylamino)-2-methyl-5-oxopentanoate (MDMO), an environmentally friendly solvent, has proven its capability to restore PVDF–UF membranes that have reached the end of their lifespan. The schematic illustration of the conducted study is shown in Figure 1. The choice of the eco-friendly solvent was made based on its affinity for the membrane matrix, as elucidated by the Hansen solubility parameter theory, which considers the interactions of van der Waals, polar, and hydrogen bonding interactions (Figure 1a) [47]. Following the treatment with a green solvent, the water permeability of the PVDF membrane increased from 47.6 ± 4.7 L m^−2^ h^−1^ bar^−1^ to 390.9 ± 8.2 L m^−2^ h^−1^ bar^−1^, surpassing the performance level of a fresh membrane (Figure 1c). Additionally, the membrane maintained a consistent rate of pollutant retention. The utilization of MDMO is reported to cause PVDF material breakdown owing to the presence of polar organic solvents. The dissolution process was significantly affected by the degree of moisture (wetness) and the temperature at which it was administered. The PVDF membrane demonstrated a high level of stability when exposed to MDMO, even in its wet form. However, it is important to be aware that raising the temperature may lead to membrane disintegration, as maintaining a temperature of around 25 °C allowed the elimination of irreversible fouling, thus helping in the restoration of membrane performance.

Tian et al., in a separate study, devised an advanced three-step procedure to revive the PVDF–UF membrane at the end of its lifespan [48]. This procedure included cleaning (i.e., immersing membrane in 0.5% sodium hypochlorite (NaOCl) solution for 2 h followed by membrane soaking in 1.5% citric acid for 2 h), solvent treatment (MemReg^®^, primary consisting of dimethyl sulfoxide (95%) and ethanol (5%)), and hydrophilic modification (using dopamine hydrochloride dissolved in 10 mM Tris-HCl buffer solution (pH = 8.5)). The surface hydrophilicity of the membrane was significantly improved by introducing hydrophilic alteration since the PVDF membrane had become very hydrophobic as a result of regular washing with chemical agents. The technique has been effectively expanded to regenerate a designed UF membrane module at the end of its lifespan, which has an effective filtration area of 1600 m^2^. The regenerated membrane module exhibited a substantially reduced trans-membrane pressure in comparison to the control end-of-life membrane module, even when operated at a decreased water production rate. Deqian et al. demonstrated the regeneration of UF and RO membranes using both physical (foam ball swabbing, water rinsing, gas–liquid cleaning, and ultrasonic treatment), chemical (using detergents, complex compounds, and oxygen-reducing agents), and combined approaches [49]. The cleaning mechanism resulted in >99% of membrane rejection performance recovery. Ponti et al. demonstrated an effective lithium–calcium selective separation using 10-year-old NF90 membranes. The authors immersed the end-of-life membrane in 1% sodium bisulfite for a long period prior to its utilization in a laboratory-scale setup [50]. Although some studies have shown successful lab-scale demonstrations for membrane regeneration, less attention has been given to regenerating PA-based membranes. The development of a technologically viable method to restore their functionality has been hampered by an inadequate comprehension of foulant properties, their interaction with the membrane, and associated high costs.

### 2.2. Polymeric Membrane Upcycling

Upcycling techniques refer to the process of recycling membranes that are nearing the end of their lifespan in order to create new membranes with improved sieving accuracy using chemical modifications. Currently, there is significant research and the implementation of upcycling technology for repurposing discarded MF/UF membranes into reclaimed NF membranes [51]. The key to upcycling these membranes is the effective implementation of interfacial polymerization (IP) on the surface of MF/UF membranes that have reached the end of their lifespan [52]. Dai et al. demonstrated the direct application of IP (occurred via soaking the membrane in 0.05 wt/v% piperazine aqueous solution for 2 min followed by 0.04 wt/v% trimesoyl chloride in hexane for 30 sec soaking) on used MF membranes to obtain recycled NF membranes with satisfactory performance (i.e., rejection 80–90% against Na_2_SO_4_) [53]. It is important to note that the effectiveness of this direct IP relies on the consistency and water-attracting properties (hydrophilicity) of the used MF membrane surface, which can be influenced by the material of the substrate membrane and the distribution of fouling on the surface [54,55]. The deposited foulant over the worn-out membrane turned out to be beneficial for water transport through the upcycled NF membranes. The authors ascribed this to the gutter effect that expanded the water transport area of the PA layer [56]. Conversely, the presence of foulant within the pores of used MF membranes led to an increased water transport resistance in repurposed NF membranes, thus reducing permeate flux [53]. As the fouling deposition on MF/UF membranes may differ depending on operational circumstances and the type of water being treated, a more adaptable cleaning–healing–IP upcycling technique was introduced [57]. The cleaning process (i.e., exposing the membrane to 0.5% NaOCl solution for 2 h followed by it soaking to 2 wt% oxalic acid for 2 h) accounted for contaminant removal from the membrane surface, while the healing process carried out via polydopamine (2 g/L dissolved in 10 mM Tris-HCl buffer solution) layer deposition guaranteed the formation of a consistent and water-attracting interface for the subsequent IP process.

**Figure 2:**
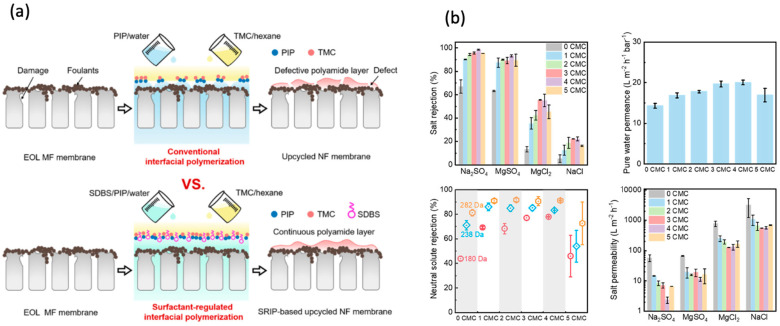
Polymeric membrane upcycling: (**a**) schematic mechanism; (**b**) upcycled membrane permeance/rejection performance [54]. Reprinted with copyright permission.

It has been reported that tannic acid (TA) and polydopamine (PDA) can be utilized as effective restorative agents to promote the seamless formation of the PA layer and to enhance the IP reaction [57,58]. The fast polymerization rate of TA and its low cost compared to PDA makes it more appealing for upcycling operations at a larger scale. The tannic-acid–iron (TA-Fe) healing-based three-step procedure was used to upcycle MF membranes. The produced NF membrane demonstrated a rejection of 96.9% against Na_2_SO_4_ with 23.7 ± 1.4 L m^−2^ h^−1^ bar^−1^ water permeance [58]. A new surfactant-controlled IP method has been recently reported to simplify the procedure down to a single stage (Figure 2). This is accomplished by introducing the aqueous ingredient sodium dodecylbenzene sulfonate (SDS), which aids in the effective absorption of the amine monomers [54]. The schematic illustration of the fabrication mechanism is depicted in Figure 2a. Costs are kept to a minimum while a high level of separation performance is achieved. The technique effectively minimizes economic expenses while achieving a high separation performance (i.e., Na_2_SO_4_ rejection rate of 98.6 ± 0.4%), as shown in Figure 2b. Somrani et al. demonstrated the transformation of an end-of-life RO membrane into a cationic exchange membrane for its application in fungal microbial fuel cells. The fabrication was conducted using two successive steps: (1) degrading the PA layer via chlorination and (2) deposition of polystyrene sulfonic acid electrolyte solution. The prepared membrane was characterized via several methods to determine transference number, diffusion flux measurement, cationic exchange capacity, and other properties. The fabricated membrane showed low external resistance (8 k Ohm) during four days of the experiment (carried out in a fungal microbial fuel cell laboratory setup) when compared with the Nafion© 117 (37 k Ohm), the usual cationic membrane [59]. Although the viability of upcycling and its mechanism has been demonstrated, the procedure still encounters some obstacles. Due to potential inconsistencies between the module types of MF/UF membranes and NF/RO membranes, it is necessary to perform additional disassembly and reassembly processes throughout the upcycling process. If the prospective MF/UF membrane is of the spiral-type module, the upcycling process is highly advantageous. Alternatively, the upcycling process requires more intricate and time-intensive operations. The aforementioned upcycling approach may be readily implemented for UF membranes that have reached the end of their lifespan, as the surface of UF membranes is more suited for an IP reaction compared to MF membranes [60]. However, the upcycled membrane is presently not as effective for fabricating the RO membrane due to the requirement of distinct surface structure and characteristics needed for m-phenylenediamine-based PA layer creation as it differs from the piperazine-based PA layer. Thus, further efforts are required to overcome the technological obstacle of upcycling MF/UF membranes at the end of their lifespan in order to produce recycled RO membranes.

### 2.3. Polymeric Membrane Downcycling

**Figure 3:**
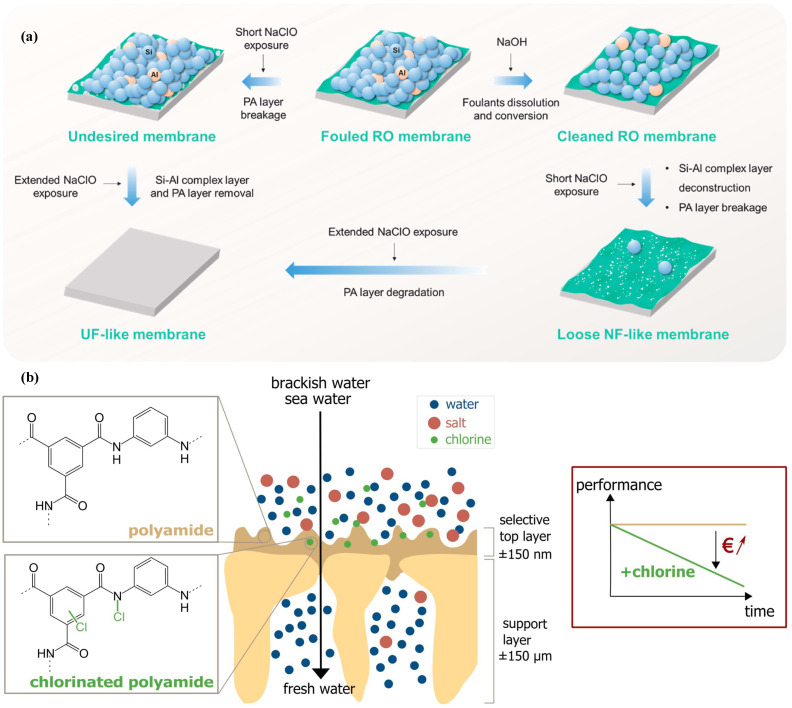
Membrane downcycling: (**a**) schematic illustration of RO membrane downcycling to NF and UF [71]; (**b**) PA layer chlorination mechanism [72]. Reprinted with copyright permission.

Downcycling refers to the transformation of end-of-life membranes to directly prepare recycled membranes with lower sieving accuracy. It is considered the most convenient method when it comes to recycling NF/RO membranes. As fouling removal from the PA surface of the end-of-life NF/RO membranes is challenging owing to its complex nature and sensitive surface properties of the membrane, peeling the PA layer via chemical oxidation is considered an easy and cost-effective approach for fouling removal and membrane recycling. Subsequently, the transformed membrane could be utilized as an MF/UF element for pretreatment of the feed or for non-potable water reuse applications (Figure 3a). The primary approach involved in membrane downcycling is the partial or full destruction of the PA layer using oxidizing agents such as NaOCl [37], potassium permanganate (KMnO_4_) [61], hydrogen peroxide (H_2_O_2_) [62], and their mixture [63]. The utilization of NaOCl is perhaps more common for NF/RO membranes downcycling due to the susceptibility of PA to NaOCl, causing its degradation [22,64,65,66]. Paula et al. reported NaOCl as a better oxidizing agent when performing oxidative treatment on end-of-life RO membranes using KMnO_4_ and NaOCl. The membrane downcycled using NaOCl (pH 11) demonstrated 27–39 times enhancement in flux when compared to the pristine RO. In contrast, a 22–29 times increase was observed when using KMnO_4_ as an oxidizing agent [67]. This is based on the mechanism of N-chlorination and hydrolysis of the amide bond, consequently causing oxidative degradation to the PA layer upon exposure to NaOCl (Figure 3b) [68,69]. Several studies have demonstrated the downcycling of the RO membrane to NF, UF, and MF membranes and NF membrane to UF and MF at both lab and pilot-scale levels [37,63,70,71].

The downcycling process is primarily controlled by selecting an appropriate concentration and duration of NaOCl for membrane exposure (i.e., choosing the suitable NaOCl attack strength, measured in ppm × h) [73]. When the exposure strength of NaOCl ranges from 1000 to 48,000 ppm × h, the treated RO membranes displayed characteristics similar to those of NF membranes, whereas when reaching 200,000 to 350,000 ppm × h, the water permeance exceeds 100 L m^−2^ h^−1^ bar^−1^, and the salt rejection rate reaches less than 15%, gradually approaching the rejection range of UF membranes [74,75]. Another benefit of NF/RO membrane downcycling is their direct utilization for end application. Most commercial NF/RO membranes are spiral wounds and are packed with glass fiber casing to provide the mechanical support needed during high-pressure operation. Therefore, the end-of-life NF/RO membranes can be readily downcycled without the need to alter the type of membrane modules [66]. Pacheco et al. demonstrated a novel gravity-driven housing design for NF and UF membranes downcycled from two RO membranes [76]. To monitor the performance, two housing devices were developed: one featuring simple fitment and the other featuring an advanced end-cap design. The testing was performed across a spectrum of conditions, including submerged, non-submerged, continuous, and intermittent. The recycled NF membrane exhibited low salt removal and permeability (~1.7 L m^−2^ h^−1^ bar^−1^) but showed high rejection (>81%) against dissolved organic carbon, whereas the recycled UF membrane maintained its biopolymer removal (>74%) with an 18% fold higher permeate flux value compared to NF.

A six-year-old Filmtech LC-LE-4040 PA membrane was downcycled to UF by performing acid and basic cleaning using 0.1% sodium hydroxide and 2% citric acid, respectively, and exposing the membrane to NaOCl solution followed by its performance evaluation using different feed types (i.e., tap water, wetland permeate, and membrane bioreactor effluent) [37]. The recycled membrane showed an average chemical oxygen demand (COD) removal of 65% and high flux recovery. Seibel et al. demonstrated the treatment of brackish and surface water using downcycled RO membranes. The authors rated the obtained performance to NF when performing the separation test using a downcycled RO membrane (i.e., obtained after 10,000 ppm × h NaOCl exposure). The oxidative treatment resulted in 3.1× permeability and 2.42× roughness enhancement, whereas the selectivity of the membrane against salt and acetaminophen decreased by 4.35× and 1.5×, respectively [77]. Paula et al. performed an environmental and economic evaluation of the recycled end-of-life RO membranes. The TFC-RO membrane, when subjected to NaOCl solution, exhibited comparable performance and characteristics to porous membranes. According to the authors, substituting a new UF membrane spiral element, which typically lasts about 5 years, with a recycled membrane that is expected to last around 2 years might lead to a cost reduction of 98.9% in water treatment expenses. The researchers’ study employed the applied material input per service unit method, which demonstrated that the chemical conversion of a single 8-inch spiral element of an end-of-life RO membrane weighing 13.5 kg yields a total of 2609.81 kg of materials that are neither withdrawn from the environment nor polluted. The most significant finding of this study is that the use of recycled membranes may result in economic advantages accompanied by environmental benefits [78].

### 2.4. Polymeric Membrane Re-Preparation

**Figure 4:**
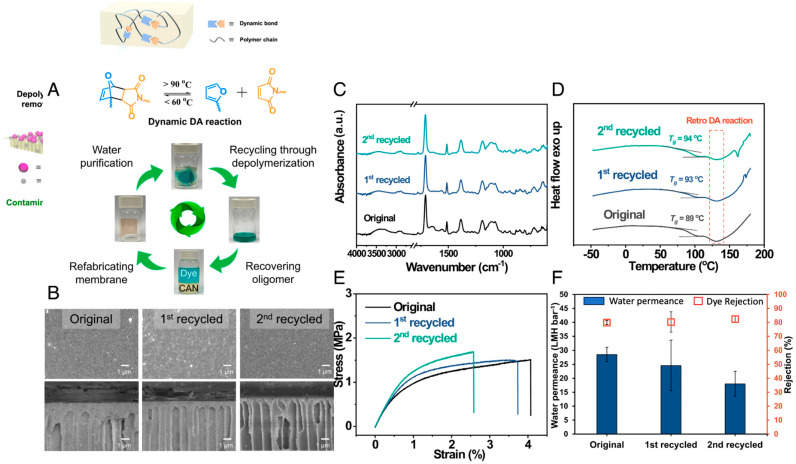
Membrane re-preparation: (**A**,**B**) membrane morphology and its fabrication schematics; (**C**–**F**) membrane characterizations [79]. Reprinted with copyright permission.

The term re-preparation refers to the complete deformation of end-of-life membranes and refabrication of the same or different membrane types using the recovered material. This is performed by dissolving the membranes in organic solvents and then utilizing the resulting solution to produce new membranes using recycled polymer. This method is especially well suited for membranes that experience substantial damage, which greatly impairs their ability to selectively separate substances. Li et al. reported a novel, high-performing, sustainable membrane that can be recycled in a closed-loop system after extended periods of use for water purification (Figure 4). The process included combining membrane technology and dynamic covalent chemistry to create thermally reversible covalent adaptive networks with furan–maleimide Diels–Alder adducts. These networks were then used to produce integrally skinned asymmetric membranes using the nonsolvent-induced phase separation approach (Figure 4A,B). The closed-loop recyclable membranes demonstrated exceptional mechanical properties, thermal and chemical stabilities, and separation performance due to the stable and reversible characteristics of the covalent adaptive network (Figure 4C–F). These properties were comparable to, or even surpass, those of the most advanced nonrecyclable membranes. Furthermore, the utilized membranes exhibited the ability for closed-loop recycling, which allowed them to maintain consistent separation performance and properties via depolymerization (when heated at 140 °C in dimethylformamide (DMF) solution) to eliminate contaminants and subsequent refabrication into new membranes via Diels–Alder adduct dissociation and reformation. The conducted work had the potential to address the deficiencies in the closed-loop recycling of membranes and stimulate the development of environmentally friendly membranes for a sustainable membrane industry [79].

Wei et al. reported a one-step fabrication method for polyimide NF membrane with high stability and rejection [80]. The process included the creation of a soluble polyimide polymer containing both imide rings and flexible pendant groups by tailoring its chain structure. The NF membranes were fabricated via a phase inversion process, where the polyimide polymer served as the matrix and resulted in an integrally skinned asymmetric design. The acquired membrane demonstrated high flux and rejection against dye, polyethylene glycol, and typical salts. In addition, the complete dissolution of polyimide membrane in DMF, a commonly used solvent for fabricating the membranes, and stable membrane performance even after four cycles of re-preparation clearly illustrated its potential for sustainable long-run operation. Pontie et al. utilized polypropylene spacers of end-of-life RO membrane for fabricating the flat-sheet MD membranes using the classical phase inversion conducted in four steps: (1) dissolution of both polypropylene (60%) and low-density polyethylene (40%) in pure xylene; (2) immersion in methanol (10–15 mints, 40 °C); (3) drying in an oven (20 mints, 60 °C) and ambient condition (18–24 h); and (4) oven drying (1 h, 60 °C) before conditioning. The potential of the manufactured membrane for MD application was demonstrated by the congruence of the obtained properties [50]. Nevertheless, the reusability of polymer can significantly enhance the sustainability of polymeric membranes [81]; the utilization of biodegradable polymers as substitutes is advised owing to the significant carbon emissions linked to polymer manufacturing derived from fossil fuels [82].

In addition to the re-utilization of recovered polymer for fabricating the membranes, the use of the recovered polymer for applications such as adsorption/absorption is also reported. Patel et al. obtained PVDF polymer in the shape of spherical beads (from membranes that were nearing the end of their useful life by utilizing DMF as a solvent) and tested their application feasibility for methylene blue decolorization from the aqueous stream [83]. Liang et al. demonstrated the Fe^3+^ determination potential of carbon dots derived from the pyrolysis of discarded RO membranes [84]. The pyrolysis process conducted at 600 °C resulted in 28 wt% oil, 17 wt% non-condensable gases, and 22 wt% char production. While the re-preparation of polymeric membranes at the end of their life cycle is the best-suited pathway for sustainability inclusion in membrane technology, it may incur high costs and encounter implementation difficulties at the site. Moreover, the utilization of organic solvents for polymer dissolution is incongruent with the principle of sustainability [51]. Therefore, it is strongly advised to utilize green solvents, which have less environmental effects (i.e., non-carcinogenic or toxic, biodegradable and non-accumulative, possess less potential for ozone layer depletion and greenhouse gas emissions, and exhibit negligible other adverse environmental effects) compared to traditional solvents, for the dissolution of damaged end-of-life membranes in order to reconstitute them [85].

## 3. Membrane Fabrication Using Recycled Waste

Polymeric membranes are generally manufactured using a variety of monomers/polymers, including polystyrene, PSF, PES, polyaniline, PVDF, and others. The industrial manufacturing of these chemical compounds causes significant greenhouse gas emissions. In addition, the application of these monomer/polymer compounds in daily necessities has been posing a massive burden for their post-utilization disposal. The emergence of waste and its recycling potential has attracted attention to its application in membrane fabrication [42,86,87,88,89]. The utilization of recycled waste for fabricating the membranes can help in reducing the environmental impact by 2× amount (i.e., eliminate the use of polymer for membrane fabrication and its associated environmental impact and mitigating the effect of waste on the environment via its utilization), thus helping in maintaining environmental sustainability. The below sections describe the potential waste sources that can be used for fabricating the membrane and provide a summary of the works conducted to make the membrane process sustainable.

### 3.1. Recycled Polystyrene

Polystyrene is a polymer often employed in the form of foamed polystyrene known as styrofoam, composed of 2% polymer and 98% air [90]. Styrofoam is primarily used in disposable utensils production, building insulation, making food packing containers, packing delicate items, etc [91,92]. Annual production of approximately 14.7 and 6.6 million metric tons (MMT) in 2016 was reported for polystyrene and foamed polystyrene, respectively [93]. The passage of time might have enhanced its utilization and subsequent annual waste generation. Unfortunately, post-consumer waste is disposed of in the municipal landfill, creating a substantial waste quantity and posing an environmental burden. Additionally, most plastics take a considerable length of time to decompose, often lasting several hundred years [94].

**Figure 5:**
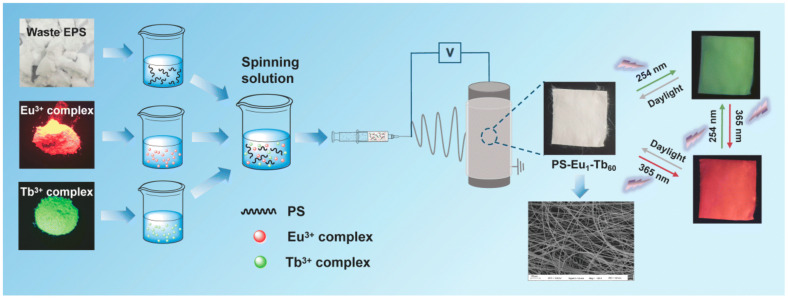
Waste expanded-polystyrene-derived membrane fabrication [88]. Reprinted with copyright permission.

Ghaly et al. carried out a comparative study using two polystyrene wastes (i.e., transparent and white colors derived from the used yogurt packaging cups) and DMF as a solvent to fabricate polymeric membranes while using membranes fabricated via commercial polystyrene pellets as a control. The utilization of both polymer materials, regardless of their transparency or white color, does not significantly deviate from commercial polystyrene membranes when fabricated using the nonsolvent-induced phase separation (NIPS) technique. The results demonstrated that the polystyrene membranes synthesized from both sources had comparable overall characteristics and thus could be effectively used for the production of flat-sheet membranes [42]. Fan et al. reported UV-excited dual-mode multicolor luminescent electrospun fiber membrane fabrication using waste-expanded polystyrene (2.5 g dissolved in 2.5 mL DMF) by co-doping with a Europium (Eu^3+^) complex and terbium (Tb^3+^) complex via electrospinning (Figure 5) [88]. Under UV light excitation, the luminescence analysis results indicated that all the as-prepared fiber membranes with varying mass ratios of the two complexes could emit Eu^3+^ ions and Tb^3+^ ions characteristically. As illustrated in Figure 5, the fiber membranes exhibited strong visible luminescence of different colors when exposed to 254 nm and 365 nm UV light. Ramos-Olmos et al. manufactured membranes via phase inversion using foamed polystyrene, N-methyl-2-pyrrolidone (NMP), and PEG and dioctyl phthalate as polymer, solvent, and additives, respectively [95]. The presence of additives altered the modified membranes’ surface properties which subsequently resulted in greater water permeability compared to the normal polystyrene used as a control.

The use of recycled polystyrene with functionalized carbon nanotubes and DMF (solvent) for fabricating UF membranes to treat river water was also reported by Adamczak et al. [96]. The results obtained indicated that pristine polystyrene membranes possessed comparable surface properties to membranes composed of other polymers and could be utilized indiscriminately with them. The outcomes achieved via surface water purification exhibited similarities to those documented in other studies employing commercial membranes. The membranes have shown a significant capacity to extract phenolic chemicals, suggesting a strong potential for effectively eliminating these pollutants. Ke et al. fabricated hydrophobic electrospun polystyrene nanofibrous membranes (fabricated using four polystyrene solutions of varying concentrations prepared in MDF) for desalination utilizing the DCMD configuration [97]. The developed membrane effectively desalinated saltwater, showing over 99.99% rejection. The water flow through the membrane dropped as the membrane thickness increased and the pore size decreased under the applied conditions for all types of feed solutions. Huan et al. created electrospun polystyrene fibers by dissolving polystyrene beads in a combination of DMF and tetrahydrofuran [98]. It was observed that augmenting the ratio of tetrahydrofuran to DMF led to the production of more robust and smoother fibers without any irregularities. This phenomenon was attributed to the creation of a polymer solution with higher viscosity as a result of the increased proportion of these solvents. The authors further examined the impact of the electrospinning voltage on the synthesis process and observed enhancement in generated mat hydrophilicity when changed from 12 to 20 kV. The microscopic analysis of the manufactured fibers revealed the presence of intertwined fibers arranged in a strand-like configuration, which was identified as the underlying cause for the observed high tensile strength of 1.5 MPa. Although the efficacy of the fiber mats for water treatment was not examined by the authors, their work yielded significant findings that can be highly advantageous in the development of recycled polystyrene membranes.

### 3.2. Recycled Polyethylene Terephthalate

Polyethylene terephthalate (PET) is one of the most widely used polymers. PET has been used in various applications due to its chemical stability in acids and organic solvents, remarkable mechanical properties, high transparency, and good gas-barrier resistance [99]. The PET bottles can potentially be used for fabricating the membranes [100]. Such repurposing of this waste not only helps to decrease the waste of plastic bottles but also reduces polymer consumption, associated greenhouse gas emissions, and membrane production costs [101]. The use of PET to fabricate a non-woven support layer of electrospun fiber [102], ion tracked-etched porous membrane for making asymmetric multifunctional heterogeneous structures [103], mechanically stable braid-reinforced fiber membranes [104], and several membrane types are reported by the authors [105,106,107]. The use of unmodified PET for fabricating the membranes demonstrated poor mechanical properties. However, the addition of PEG with higher molecular weights resulted in higher permeation flux and a stronger structure [108]. Using recycled PET as polymer and phenol, m-cresol, or dimethyl sulfoxide as a solvent, Kusumocahyo et al. fabricated UF membranes via phase inversion. Their results showed that lowering the polarity of the nonsolvent increased water permeation. The constructed membrane also demonstrated better flux when concentrations and the molecular weight of the additives in the casting solution increased [109].

**Figure 6:**
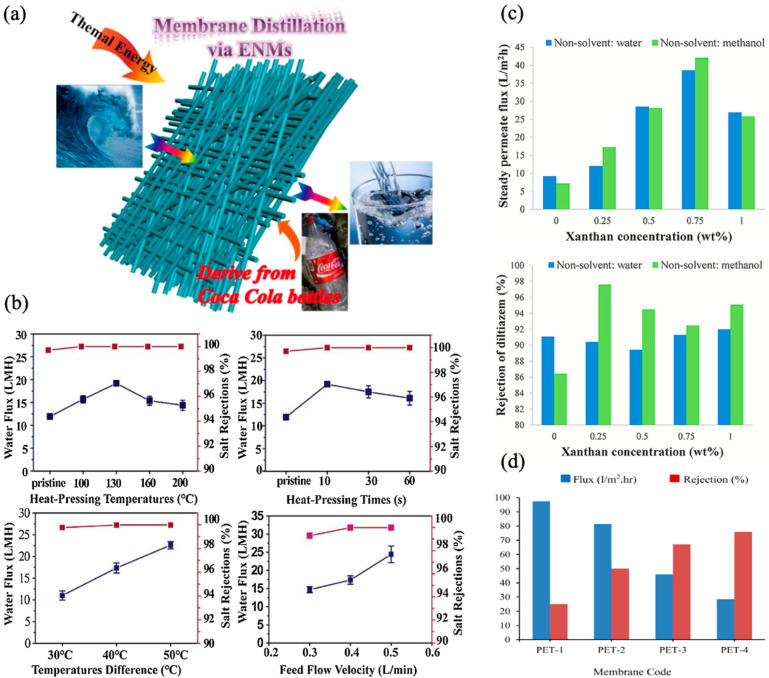
Recycled PET-derived membrane: (**a**) schematic illustration; (**b**) water flux (blue line) and rejection (pink line) at different heat pressing temperatures (10 s duration), time (at 130 °C, ΔT = 40 °C, velocity = 0.4 L min^−1^), temperature difference (feed velocity = 0.4 L min^−1^), and feed flow velocity (ΔT = 40 °C) [112]; (**c**) flux and diltiazem rejection of PAT/XA membrane [114]; (**d**) flux and humic acid rejection of recycled PET/polyvinylpyrrolidone membranes [101]. Reprinted with copyright permission.

Fabrication of the UF membrane was demonstrated in another study using recycled PET bottles as polymer, phenol as a solvent, PEG as an additive, and water/ethanol as a coagulation nonsolvent [110]. An increase in porosity and hydrophilicity was observed when the PEG concentration changed, although the pore size of the membrane was reduced. These changes brought about an increase in pure water flux and BSA solution rejection. Zander et al. fabricated an electrospun nanofiber MF membrane using recycled PET (dissolved in Hexafluoro-isopropanol and tri-butylammonium chloride) for latex beads separation (ranging from 30 to 2000 nm) [111]. The prepared membrane demonstrated 99% bead separation with only gravity filtration. Xu et al. obtained a hydrophobic (contact angle ≥ 130°) electrospun nanofiber membrane for MD when using recycled Coca-Cola bottles (dissolved in trifluoroacetic acid) and performed fluorination (Figure 6a). As shown in Figure 6b, the prepared membrane demonstrated higher flux and rejection (11–23 LMH and higher rejection, almost 100%) when compared to pristine (10 LMH and 95%, respectively) [112]. Excellent resistance toward acidic and oxidative agents in the presence of solvents such as DMF at a high temperature (100 °C) was observed by Pulido et al. when performing PEG/water and PEG/DMF filtration using recycled PET-derived UF membranes. This reflects the potency of recycled PET-derived membranes in applications where high temperature and corrosive materials are present [113].

Using PET/XA (Xanthan gum) and trifluoroacetic acid with two nonsolvent solutions, methanol and water, Kiani et al. fabricated an NF membrane to demonstrate diltiazem removal from an aqueous solution. The addition of XA (0.25–0.75 wt%) into the casting solution enhanced membrane porosity, thickness, and hydrophilicity, although further enhancement in XA (1 wt%) affected the membrane properties adversely [114]. Adding XA made the membrane structurally weaker, as indicated by tensile and elongation tests. Membranes prepared with water and methanol with XA of 0.75 wt% had the highest steady flux of ≈38 and 42 LMH, respectively. Nevertheless, the maximum rejection for diltiazem (i.e., 98% and 92%) was seen when using a PET membrane with 0.25 wt% XA dissolved in methanol and 1 wt% XA dissolved in water, respectively (Figure 6c). Utilizing the thermally induced phase separation (TIPS) method, Arahman et al. fabricated a UF membrane using recycled PET. The addition of polyvinylpyrrolidone (5 wt%) produced a sponge-like structure in the membrane sublayer, while casting without evaporation resulted in a more porous membrane. Although membranes without PVP had higher flux (97 LHM), better HA rejection (76%) was obtained when adding the 5 wt% PVP (Figure 6d). This study highlights the potency of recycled PET membranes for surface water treatment; however, further refining of the synthesis and modification processes is still required to produce high-performing membranes [101].

### 3.3. Recycled Polyvinyl Chloride

Polyvinyl chloride (PVC) is another widely used material known for its extended durability and favorable characteristics in terms of mechanical, electrical, chemical, and thermal resistance. PVC finds use in several sectors, such as building and construction, automotive, pipeline, cable industries, and the manufacturing of numerous home items. The PVC material possesses exceptional strength, durability, lightness, and versatility, rendering it an ideal choice for a multitude of applications. Several studies have utilized neat PVC for fabricating the membranes owing to its extraordinary characteristics [115,116,117,118]. However, less attention has been paid to making the membranes using waste PVC. The feasibility of utilizing waste PVC as a polymer source for fabricating the UF membrane was established. Prior to the phase inversion process being employed to produce the membrane, PVC waste acquired from post-consumer disposal was dissolved in DMF. Subsequently, different concentrations of cellulose acetate (CA) were employed to minimize the hydrophobic characteristics of PVC. The PVC UF membrane created from waste plastic achieved a BSA rejection rate of 91%, which is comparable to other PVC membranes made from commercially available precursors. The pure water flux and flux recovery ratio showed an upward trend as the CA concentration rose from 0 to 5 wt%. The pure water flux rose from 36 L m^−2^ h^−1^ to 85 L m^−2^ h^−1^, while the flux recovery ratio increased from 56% to 78%. These results suggest that neat PVC with high hydrophobicity negatively affected water permeability and anti-fouling capabilities [119].

**Figure 7:**
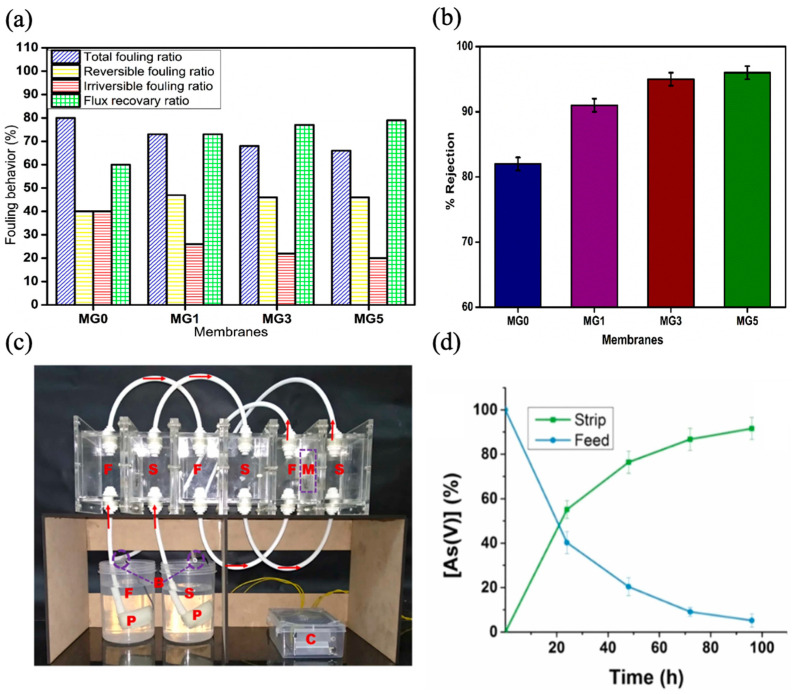
Recycled PVC membranes performance: (**a**) flux recovery ratio; (**b**) humic acid rejection (MG0, MG1, MG3, and MG5 refers to the membranes fabricated with different gum Arabic concentrations ranging from 0–5%, respectively) [120]; (**c**) prototype device used for experiments (where F = feed solution; S = strip solution; M = membrane; P = pump; C = controller; B = feedback of feed and strip solution; (**d**) percent arsenic transport [121]. Reprinted with copyright permission.

In a separate study, Aji et al. investigated the use of waste PVC as a membrane substrate and the enhancement of its hydrophilicity via the incorporation of gum Arabic, a sustainable biopolymer, for the removal of natural organic matter in water. The researchers employed the phase inversion method to prepare the membranes while adjusting the proportion of gum Arabic in a solution containing PVC and N-methyl pyrrolidone. The interactions between waste PVC and gum Arabic at various loadings (1–5% wt.%) were analyzed using a variety of analytical techniques. The GA-incorporated PVC membrane demonstrated a 25% increase in its hydrophilicity. The water permeation improved from 51 L m^−2^ h^−1^ to 98 L m^−2^ h^−1^, accompanied by a flux recovery ratio of around 80% and a rejection rate of roughly 96% (Figure 7a,b). Additionally, the onset degradation temperature saw an increase of around 30 °C, while the mechanical strength was strengthened by approximately 1.2 MPa [120]. Govindappa et al. demonstrated the integration of recycled PVC material as a base polymer and benzalkonium chloride (BAC) as an extractant to develop low-cost polymer inclusion membranes for extracting arsenic from water [121]. The polymer inclusion membrane, composed of 50% PVC dissolved in tetrahydrofuran, 40% BAC, and 10% Dioctyl phthalate, had the best transport efficiency for arsenic among membranes with varying compositions. The membrane facilitated the arsenic transport from an aqueous solution (pH = 7) to a 2 M NaCl stripping solution and showed 57.8% efficiency during a 24 h period. The membrane demonstrated exceptional transport efficiency for various anions found in water at lower concentrations when replacing 10% recycled PVC with base polymer. Nevertheless, a decrease in efficiency was noted at higher concentrations. The membrane exhibited excellent resistance to biofouling. To ensure a continuous conveyance process, a prototype capable of operating without any intervention is developed (Figure 7c); its efficiency was estimated to be approximately 91% over the course of 100 h (Figure 7d). Following five cycles, the device maintained an overall efficacy of over 80%. The findings indicate that the stability and performance of the pure PVC membrane were not influenced by the incorporation of recycled PVC, in contrast to the standard polymer inclusion membrane that did not contain recycled PVC.

### 3.4. Recycled Tire Rubber

Tire production utilizes approximately 70% of the rubber produced globally. Considering that tires contain about 60 percent natural and synthetic rubber, an estimated 6 million tons of refuse tires are produced annually. The annual global quantity of waste tires disposed of is close to 800 million (10 million tons) [122]. In an effort to achieve the objectives of resource recycling and environmental protection, developed nations have paid considerable attention to the ubiquitous use of discarded tires. Landfilling, incineration, pyrolysis, and devulcanization/reclaiming of waste rubber products have been suggested as potential solutions to the recycling and disposal issue associated with rubber waste. Since 1999, the disposal of tire waste in landfills has been prohibited by the European Commission. Out of the many methods considered, abandoning the material is the least desirable. Beyond that, the value added is negative, and this circumstance does not contribute to the product’s value. While it is possible to mitigate the hazardous pollution issue caused by refuse tires by utilizing them as a fuel source, this approach is not environmentally preferred. For instance, the incineration of waste rubber products generates secondary pollutants, including noxious odors and hazardous vapors, which contribute significantly to air pollution. Furthermore, the enormous quantity of CO_2_ produced when waste rubber is incinerated contributes to global warming. Devulcanization and rubber waste reclamation are thus the most beneficial method for resolving the disposal issue. Devulcanization and reclamation serve the dual purpose of safeguarding habitats and conserving the nonrenewable petroleum resources that provide the basic materials for these processes [123,124].

**Figure 8:**
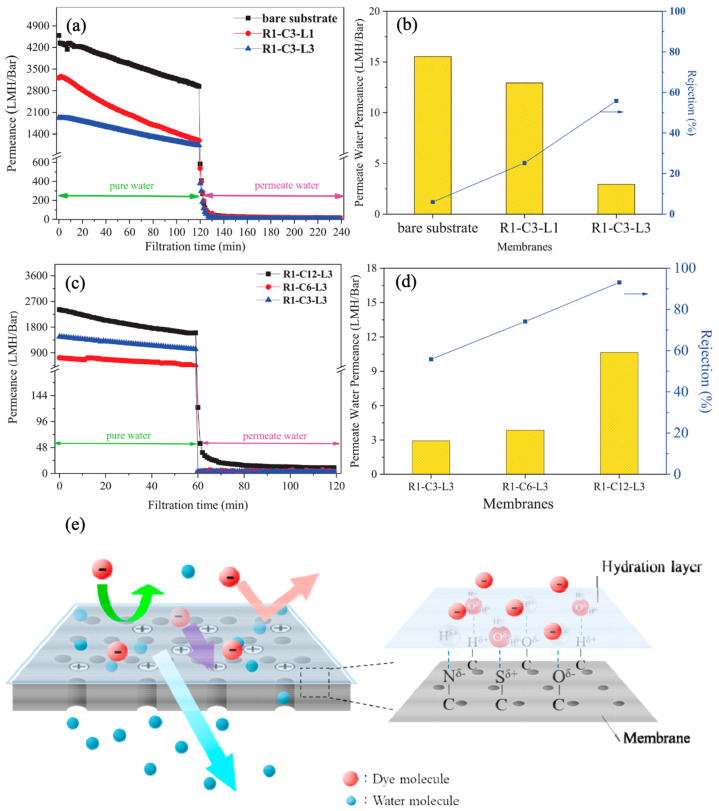
Performance of recycled tire-derived membranes: (**a**) permeance vs. filtration time; (**b**) permeate permeance and dye rejection as a function of the internal coating; (**c**) permeance vs. filtration time; (**d**) permeate permeance and dye rejection as a function of precursor concentration; (**e**) schematic representation of the separation mechanism by hydration layer [125]. Reprinted with copyright permission. (R_1_ refers to reclaimed rubber with devulcanization from a waste tire; C_3_, C_6_, and C_12_ refer to %R_1_ concentration used; and L_1_ and L_3_ refer to the number of internal coating cycles ranging from 1–3, respectively).

Using powder and devulcanized rubber derived from recycled tires, Lin et al. fabricated the composite hollow fiber NF membrane. The rubber selective layer was generated on the internal surface of the ceramic substrate subsequent to its dissolution in toluene. The layer served as a structural support, thereby enhancing the mechanical strength. The rubber casting solution, drawn into the porous substrate via the capillary effect, facilitated rubber adhesion to the substrate. The thermal and solubility properties of the rubber were predominantly influenced by the compositions of the waste rubber, as observed. The solubility of rubber powder was lower than that of devulcanized recycled rubber due to its crosslinked three-dimensional structure. An inverse relationship was observed between the number of internal coatings and permeability during filtration using MB dye solution; this relationship was attributed to the dense rubber selective layer (Figure 8a,b). Conversely, an increase in rubber concentration from 6% to 12% resulted in a corresponding rise in the permeability of purified water (from 470.2 to 1596.1 L m^−2^ h^−1^) and the permeability of the dye solution (from 3.9 to 10.6 wt%) (Figure 8c,d). This can be attributed to the augmented presence of surface hydrophilic groups, which contribute to the formation of the hydration layer (Figure 8e). The hydration layer’s anti-fouling qualities facilitated permeance recovery, surpassing 80% after washing. The hydration layer functions as a barrier to reduce the adsorption of MB molecules onto the surface of the membrane. The non-vulcanized rubber-based NF membrane, containing 12% rubber concentration and coated internally three times, demonstrated a rejection rate of 93.1% for MB. Following three filtration cycles, the membrane’s permeability remained at a level of 8.3 L m^−2^ h^−1^ while achieving a 98.1% rejection rate for MB [125]. In addition to the application of tire-derived polymeric membranes for treating the water, their utilization for gas separation is also reported elsewhere [126,127].

### 3.5. Recycled Keratin

**Figure 9:**
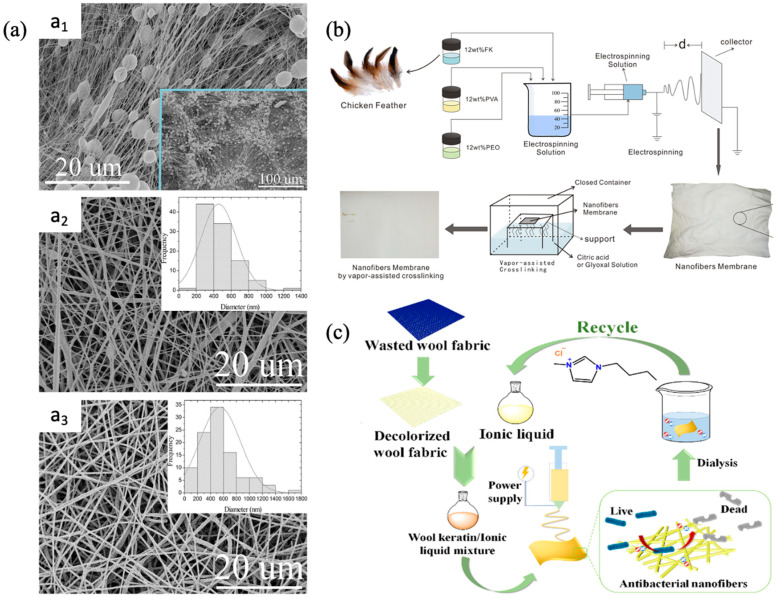
Recycled keratin-derived membranes: (**a**) morphology and diameter distribution of keratin/polyethylene oxide nanocomposite membrane ((**a_1_**–**a_3_**) refers to SEM images of 100/0 WK-PEO, 90/10 WK-PEO, and 70/30 WK-PEO nanofibrous membrane while of the inset figures depict diameter distribution of the respective membrane) [129]; (**b**) schematic illustration of electrospun membrane fabricated using chicken feather (d refers to the distance between the syringe needle and the collector) [130]; (**c**) stepwise illustration of wool fabric derived membranes [124]. Reprinted with copyright permission.

Chicken feathers, fleece, hair, hoof, and nails are the primary sources of keratin. The global accumulation of keratin refuse has steadily escalated into an unmanageable problem over time [128]. Ma et al. fabricated the wool keratin (WK)/polyethylene oxide nanocomposite membrane using waste wool fabric [129]. To enhance the spinnability of the wool keratin, the decolorized wool fabric was dissolved in formic acid containing poly(ethylene oxide) (PEO) followed by electrospinning at a rate of 0.5 mL h^−1^, a syringe-collector distance of 15 cm, and a voltage of 14 kV, for fabricating the nanofiber membranes. The prepared membrane showed average diameter enhancement from 226 nm to 379 nm, and bead formation was inhibited when 10 wt% PEO was added (Figure 9a). Ding et al. utilized keratin obtained from feathers, PEO, and PVA to fabricate membranes via electrospinning [130]. The fabricated membranes were then crosslinked using citric acid and glyoxal vapor to produce NF membranes that are suitable for filtration applications requiring resistance to solvents (Figure 9b). The mean diameter of the nanofibers exhibited an increase from about 223 nm in control to approximately 342 nm and 304 nm in the citric- and glyoxal-crosslinked membranes, respectively. This enlargement may be attributed to the diffusion of the crosslinked chains into the macromolecular chains, resulting in the formation of a crosslinking network. The process of crosslinking significantly increased the intermolecular connection of the composite membranes, resulting in higher mechanical and thermal stability.

Zong et al. reported the eco-fabrication of nanofibrous membranes with antibacterial and high moisture permeability using waste wool fabrics [131]. The NF membrane fabrication involved the blending of wool fabric keratin (dissolved in 1-butyl-3-methylimidazolium chloride) with PAN (dissolved in DMF), followed by electrospinning of the mixture. The stepwise illustration of the fabrication mechanism is shown in Figure 9c. The PAN/keratin nanofiber membrane showed significant antibacterial activity, with inhibition rates of 89.21% against *E. coli* and 60.70% against *S. aureus*, as determined by the bacterium colony count. In contrast, the PAN membrane without antibacterial capabilities did not demonstrate such effects. The moisture-management testing indicated that the PAN/keratin membrane exhibited enhanced wetting and moisture transport characteristics in comparison to the PAN membrane due to the inclusion of hydrophilic functional groups from keratin. In a separate study, the electrospinning of a polymer dope composed of a mixture of commercial PA and keratin extracted from waste goat hair was utilized to fabricate a nanofiber membrane for pigment removal [132]. Sulfitolysis was employed to extract the keratin, and formic acid was utilized to dissolve the hydrolyzed keratin to produce the keratin solution. An interconnected pore structure nanofiber membrane featuring fine, irregularly oriented beads was subsequently produced via the electrospinning of the polymer material at a rate of 0.1 mL h^−1^ and a distance of 22 cm using a 20 kV voltage. In contrast to the nanofiber composed solely of PA, the membrane composed of PA/keratin exhibited reduced crystallinity, increased porosity, and a smaller mean pore size. The reduced mechanical strength of the regenerated keratin was attributed to its rigid and stiff structure. When testing the filtration efficacy of the nanofiber membrane with PA/keratin to remove dark green anionic tannery dye, it consistently exhibited superior performance compared to the PA membrane. The highest dye rejection of 100% was achieved under acidic conditions using a 100 ppm dye solution. Nevertheless, this study did not specifically focus on the interaction and synergistic effects of PA and keratin that mutually enhanced performance. The use of keratin, extracted from the goat hair, for fabricating the NF membrane for dye removal is also reported elsewhere [133].

### 3.6. Recycled Cellulose and Its Derivatives

The use of cellulose and its derivatives for fabricating the membranes has also been reported for environmental sustainability. Studies have reported the utilization of cellulose derived from oil palm empty fruit bunches, kapok fiber, non-dyed cotton bedsheets, cotton microdust waste released during the spinning process, waste paper, and waste cigarettes for fabricating the membranes. In order to remediate dye wastewater, Teow et al. developed a cellulose/PVDF membrane using oil palm empty fruit bunches containing 40–50% cellulose [134]. The extracted cellulose was dissolved in solvents (N-Methyl-2-pyrrolidone (NMP) and N-N-dimethylacetamide (DMAc)), which was followed by lithium chloride addition. An increase in the cellulose-to-PVDF ratio in the dope led to a more rapid exchange of solvents and nonsolvents via the hydrophilic cellulose content. This was achieved by decreasing the viscosity of the dope and weakening the interaction between the hydrophobic PVDF and water. Consequently, the membrane developed wider pores. As a result of the increased solubility parameter, which accelerated the precipitation rate and encouraged the formation of a sponge-like structure, the membranes prepared with NMP solvent demonstrated greater porosity and larger pore size than those prepared with DMAc. The cellulose membrane, which was prepared using NMP solvent (cellulose-to-PVDF ratio of 96:4 and cellulose content of 3 wt%), demonstrated excellent properties such as a high permeate flux of 158.06 Lm^−2^ h^−1^ and an MB dye rejection rate of 34.9%.

**Figure 10:**
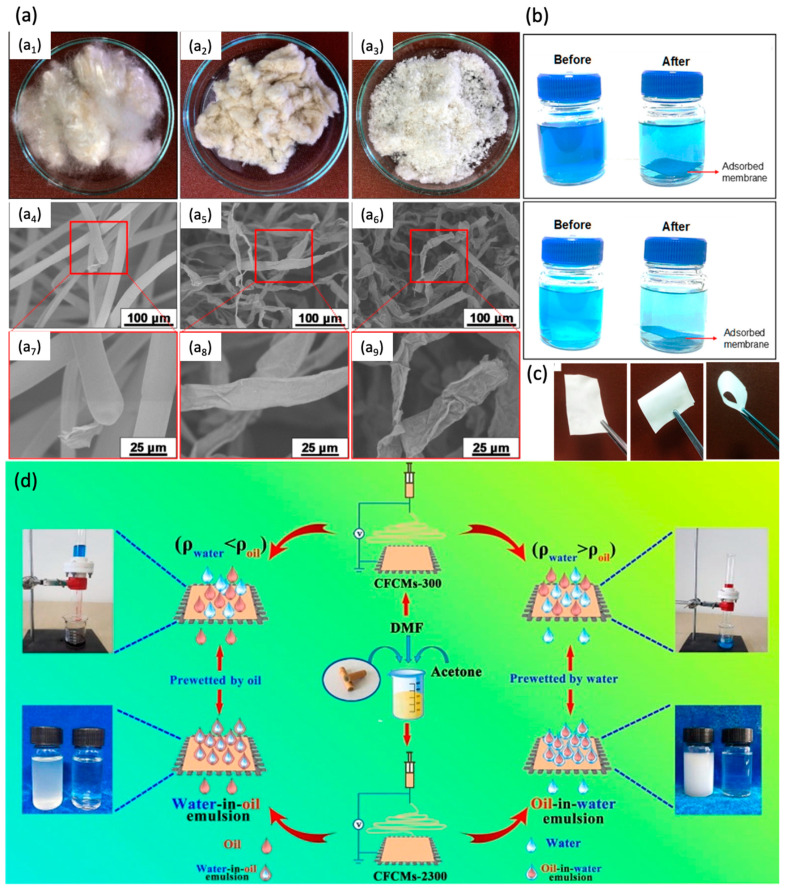
Membranes fabricated via cellulose and their derivates: (**a**) morphology of kapok fiber derived membrane ((**a_1_**–**a_3_**) refers to raw kapok fiber, cellulose microfiber after NaOH treatment, and cellulose microfiber after NaClO_2_ treatment, respectively. While (**a_4_**–**a_9_**) refer to their SEM morphologies at different resolutions); (**b**) digital photo of methylene blue before and after adsorption; (**c**) digital photos of the fabricated membrane showing [135]; (**d**) schematic illustration of CA (derived from cigarette) membrane fabrication and its performance evaluation [138]. Reprinted with copyright permission.

Mohamed et al. successfully synthesized self-assembling cellulose nanocrystal membranes by extracting cellulose nanocrystals and utilizing kapok fiber as the cellulosic source (Figure 10a) [135]. By employing the water suspension casting evaporation method, the cellulose fibers acquired via acid hydrolysis and alkali extraction of kapok fiber were incorporated into a nanoporous membrane. The pretreatment resulted in a significant increase in the cellulose content, raising it from 59.6% in the raw kapok fiber to 92.8% in the hydrolyzed cellulose microfiber. This process also led to a reduction in the size of the fiber at the micro-scale level, resulting in the formation of a nanorod or nano-needle structure (Figure 10a). By virtue of the function of surface hydroxyl groups in facilitating adsorption via hydrogen bonding or electrostatic interaction, the membrane demonstrated a highly encouraging removal efficiency of 85% when used to remove 5 ppm MB from an aqueous medium (Figure 10b). Lopatine et al. demonstrated the fabrication of UF membranes (with a 150 µm thickness) using a non-dyed cotton bedsheet as cellulose (dissolved in 1–ethyl–3–methylimidazole acetate and dimethyl sulfoxide) and observed nearly 90% polyethylene glycol (35 kDa) retention [136]. The obtained performance was comparable to the commercial and regenerated cellulose membranes. Vignesh et al. also utilized the cotton microdust released as waste during the spinning process in the textile industry to fabricate cellulose membranes [137]. The rheology and tensile experiments demonstrated good mechanical characteristics. The authors ascribed this to the presence of a small lignin amount in the membrane. The prepared membrane also exhibited a high bactericidal effect against E. coli. This was attributed to the inclusion of Zn^2+^ ions, which caused oxidative stress in the microbial cell. While the study did not specifically assess the separation performance, the physical properties and antibacterial test results make it a promising option for liquid separation due to its strong mechanical strength, resistance to bacterial fouling, and ability to attract water.

Mohamed et al. fabricated the cellulose membrane by utilizing the non-printed area of recycled newspaper and dissolving it in NaOH (7 wt%)/urea (12%) aqueous solution [139]. The resulting cellulose membrane exhibited a homogenous, symmetric, and dense structure, with mean porosity and pore size of 41.0% and 2.48 nm, respectively. However, due to its structural characteristics, the membrane exhibited low pure water permeability (0.35 m^−2^ h^−1^ bar^−1^), indicating that more fine-tuning is necessary to increase the cellulose membrane’s suitability for filtering the liquid. In a subsequent study conducted by a similar author, the addition of N-doped TiO_2_ nanorod to the newspaper-derived recycled cellulose matrix improved membrane porosity (i.e., from 41% for pristine to 64%) and hydrophilic properties. The authors ascribed this to the increase in free volume between the cellulose chain and the superficial hydroxyl group presented on the TiO_2_ surface, respectively, which subsequently enhanced the pure water flux (from 0.69 L m^−2^ h^−1^ for the pristine membrane to 4.12 L m^−2^ h^−1^ for the photocatalytic cellulose membrane with 0.5 wt% N-doped TiO_2_ nanorods). The membrane also demonstrated the efficient photocatalytic degradation of aqueous phenol under visible light (78.8%) and ultraviolet irradiations (96.6%) [140],

Using CA derived from the cigarette, an electrospun membrane for treating the oil/water emulsion was fabricated by Liu et al. [138] by dissolving the collected waste cigarette filters in a solvent mixture (10.5 mL DMF and 3.4 mL acetone). The membrane exhibited superhydrophobicity (under oil) when prewetted with the heavy oil, thus facilitating the oil permeation (i.e., >99% efficiency with a filtrate flux of >100 L m^−2^ h^−1^) while blocking the water on the other side of the membrane (Figure 10b). Doyan et al. also fabricated a CA (derived from a cigarette filter) membrane via phase inversion using DMF as a solvent to treat the oil/water emulsion [141]. The membrane demonstrated considerable potential with a permeability of 180 L m^−2^ h^−1^ bar^−1^ and a rejection rate exceeding 94% after undergoing five filtration cycles of the oil/water emulsion. Despite providing greater permeability to oil-in-water emulsions than PSF and PVDF membranes, the increased oil adhesion on the membrane surface caused the membranes to experience more severe irreversible fouling (>90.0%). The results indicated that, similar to the majority of CA membranes made from commercially available CA materials, it is necessary to optimize the membrane composition or modify the membrane surface in order to address the fouling problem.

## 4. Challenges, Limitations, and Future Work Directions

The recycling of membranes and polymers for membrane refabrication is a cost-effective and sustainable approach as the practice helps material conservation, reduces greenhouse gas emissions, and mitigates utility usage and their subsequent transformation into waste. However, it requires several prerequisites that are of great importance and have an influence on the sustainability of the overall process. For instance, in the case of membrane (i.e., MF, UF, NF, and RO) regeneration, the membrane has to undergo chemical cleaning for fouling (i.e., organic, inorganic, and biological) removal from the end-of-life membrane surface. The downgraded or upgraded membrane application could further involve PA layer degradation/removal and re-polymerization, respectively, after chemical cleaning. Similarly, the regeneration of the polymer for membrane refabrication requires membrane chemical cleaning prior to the dissolution of the polymer in a solvent or post-treatment of the dissolved mixture for polymer extraction and re-utilization. All these practices involve the immense utilization of hazardous/toxic chemicals. In addition, when combined with the foulants deposited on the membrane surface, the generated mixture becomes even more challenging.

Therefore, the wastewater produced by these operations requires special attention and careful management before being discharged into the environment. Although several studies have demonstrated the membrane recycling and re-utilization of the polymer for membrane fabrication, less attention has been paid to addressing this unavoidable challenge. Similarly, the utilization of discarded waste for fabricating the membranes is a great step for sustainability inclusion in membrane technology. However, the processing cost of these wastes, including both economic and environmental for transforming them into membranes need to be evaluated for their wide implementation. Also, the long-term performance of the recycled/refabricated membranes and membranes fabricated via recycled waste needs to the investigated to evaluate their lifespan and cost–benefit analysis. The inclusion of the life cycle perspective and carbon footprint indicators for the adopted strategies would greatly help in their practical implementations [142,143].

## 5. Conclusions

Desalination and water treatment using membrane-based separation processes is a promising pathway to combat water scarcity. Nevertheless, the immense utilization of membranes poses several challenges. When approaching end-of-life, landfill or incineration, which are conventionally utilized for discarded membrane disposal, adversely affect the environment. Also, the replacement of these membranes is cost-intensive, while the fabrication involves the use of several fossil-fuel-derived chemicals, which results in both resource depletion and greenhouse gas emissions. Parallel to this, lifestyle advancements have led to significant enhancements in solid waste generation, thus overwhelming the landfills and subsequent environmental damages. Given that, the disposal of plastic waste has become an emerging concern due to its slow degradation, occurrence in micron sizes in the aquatic and terrestrial environment, and accumulation in living organisms. These approaches are intricately linked to Sustainable Development Goal 12 of the United Nations, which endeavors to establish patterns of consumption and production that are sustainable. By reusing waste and membranes in the process of manufacturing them, not only could resources be conserved but also the environment could be safeguarded. Implementing this approach may greatly decrease the need for raw materials in the manufacturing process, as well as the environmental pollution that is linked to their production and disposal.

## Data Availability

No data was used in this study.
